# The Effect of Amnion-Derived Cellular Cytokine Solution on the Epithelialization of Partial-Thickness Donor Site Wounds in Normal and Streptozotocin-Induced Diabetic Swine

**Published:** 2009-10-20

**Authors:** Juri Bergmann, Florian Hackl, Taro Koyama, Pejman Aflaki, Charlotte A. Smith, Martin C. Robson, Elof Eriksson

**Affiliations:** ^a^Division of Plastic Surgery, Brigham and Women's Hospital, Harvard Medical School, Boston, Mass; ^b^Stemnion, Inc, Pittsburgh, Pa; ^c^Division of Plastic Surgery, Department of Surgery, University of South Florida, Tampa

## Abstract

**Objective:** The purpose of this study was to determine whether amnion-derived cellular cytokine solution (ACCS) could improve the quality of epithelialization and accelerate closure of dermatome-created partial-thickness wounds in normal and streptozotocin-induced diabetic pigs. **Methods:** Dermatome-created partial-thickness wounds were sealed with wound chambers in healthy and diabetic pigs and were injected with ACCS. Wound fluid was exchanged daily for total protein concentration, and biopsies were taken on days 6, 8, 10, and 12. Epithelialization, thickness of epidermis, number of epidermal cell layers, and rete ridges were evaluated. **Results:** The macroscopic appearance of the wounds and speed of healing was similar in all groups at each time point. All wounds were healed by day 6. The epidermis was thicker in the ACCS-treated diabetic wounds than in the controls (140.6 μm vs 82.7 μm on day 12 in diabetic pigs). There were more cell layers (13 vs 7.7) in ACCS-treated diabetic pigs on day 12. The number of rete ridges per 2.5 mm was greater on day 12 in the ACCS-treated diabetic wounds (13 vs 8). There was also a significant increase in the number of rete ridges in ACCS-treated nondiabetic pigs but no difference in epidermal thickness or number of cell layers. **Conclusion:** In diabetic pigs, we found a significantly thicker epidermis and more cell layers and rete ridges in the ACCS-treated wounds. Healthy pigs showed more rete ridges but no difference in thickness of epidermis or number of cell layers on day 12.

Stem cells or stem cell–like multipotent cells have great potential in the wound healing/tissue repair arena.^1^. They have the ability to differentiate into the various cell types of the repair process and to secrete the humoral messengers necessary to mediate the cellular processes.^2^–^4^ Adult stem-like cells derived from amnion have been shown to secrete many cytokines and growth factors.^5^–^7^

Amnion-derived multipotent progenitor (AMP) cells have been reported to increase the rate of gain of incisional breaking strength and decrease the incidence and severity of acute wound failure.^8^ It was postulated that one of the possible mechanisms for the improvement of acute wound healing in these experiments was that the AMP cells could be making the necessary cytokine cocktail at the proper time and dose to affect a trajectory shift.^8^ The secreted cytokines from AMP cells have been labeled amnion-derived cellular cytokine solution (ACCS) and studied using qualitative antibody arrays, enzyme-linked immunosorbent assay (ELISA), multiplex expression analysis, and mass spectroscopy.^9^ The solution was found to contain a combination of cytokines and growth factors at physiological levels. There was a good correlation between the levels of cytokines in ACCS and available levels of cytokines reported in the literature as measured in humans in normal and diseased states.^9^ Topical application of ACCS has been reported to accelerate wound closure of experimental chronic-infected granulating wounds.^1^

The purpose of the present study was to evaluate the effects of local application of ACCS in a wound-healing model of epithelialization. The model is a partial-thickness skin graft donor site wound.^10^ The wounds were evaluated in both normal and streptozotocin-induced diabetic swine.

## MATERIALS AND METHODS

### Amnion-derived cell solution

ACCS was prepared by Stemnion, Inc (Pittsburgh, Pennsylvania) as follows: amnion-derived cells were dissociated from the amnion of donated, full-term, scheduled C-section placentae. AMP cells, a subpopulation of multipotent cells selected from amnion-derived cells, were used to produce ACCS. Once isolated from the placenta and selected, AMP cells were grown to confluency in STM100, a proprietary cell media. Cell cultures reached confluency in 7 to 9 days, and the ACCS was harvested on days 12 and 15.^9^

### Design of the study

In the first part of the experiment, 72 partial-thickness wounds were created on the backs of 3 female healthy Yorkshire pigs. Twenty-four wounds were received treatment with 1-mL ACCS and 1-mL saline containing 200 units of penicillin and 200 mg of streptomycin placed in the wound chamber every other day (group ACCS qod). Another 24 wounds received the same amount of ACCS and saline with antibiotics but every 4 days (group ACCS q4d). The 24 control wounds received 2 mL of saline with 100 units of penicillin and 100 mg of streptomycin per milliliter. The wound fluid was processed for total protein concentration as a noninvasive parameter for reestablishment of the epithelial barrier function. Biopsies were taken on days 6, 8, 10, and 12. Specimens were fixed in 10% neutral buffered formalin and processed for hematoxylin and eosin staining. Average thickness of the epidermis and the number of cell layers were recorded.

In the second part of this study, the same size and number partial-thickness wounds were created on the backs of 3 diabetic pigs. Experimental wounds consisted of 5 groups receiving incremental dilutions of ACCS (0.445-mL ACCS/cm^2^, 0.255-mL ACCS/cm^2^, 0.111-mL ACCS/cm^2^, 0.045-mL ACCS/cm^2^, and 0.022-mL ACCS/cm^2^). Wounds treated with saline served as controls. Wound biopsies were taken on days 6, 8, 10, and 12 for histological evaluation. Wound fluid was exchanged every 24 hours and evaluated using a total protein ELISA. Biopsies were stained with H&E for evaluation of rete ridge formation and thickness of epidermis on different biopsy days.

### Animals

All animal procedures were approved by the Harvard Medical Area Standing Committee on Animals and conformed with the regulations related to animal use and other federal statutes. Female Yorkshire pigs (Parson's Farm, Hadley, Massachusetts), weighing 40 to 50 kg at arrival, were allowed to acclimatize for 1 week before initiation of the experiment.

### Induction of diabetes

Animals were weighed, received induction anesthesia with tiletamine HCl and zolazepam HCl (Telazol) (10 mg/kg BW, Wyeth Madison, New Jersey) and xylazine (2.5 mg/kg BW, Xyla-Ject, Phoenix, St Josephs, Missouri), followed by general anesthesia with isoflurane (Novaplus; Hospira, Lake Forest, Illinois) inhalation (2%–3% vol) and were weighed. Streptozotocin (Zanosar; Pharmacia, Pfizer, New York) was applied at a dose of 150 mg/kg body weight. The solution was administered through an intravenous catheter over 1 minute. Buprenorphine was administered for pain control. Serum glucose concentration was measured daily. The pigs were treated with a subcutaneous injection of short-acting insulin and long-acting insulin zinc suspension (Normulin; Novo Nordisk, Princeton, New Jersey; Humulin; Eli Lilly, Indianapolis, Indiana) to keep the blood glucose concentration between 350 and 550 mg/dL.

### Wounding

**Figure 1:**
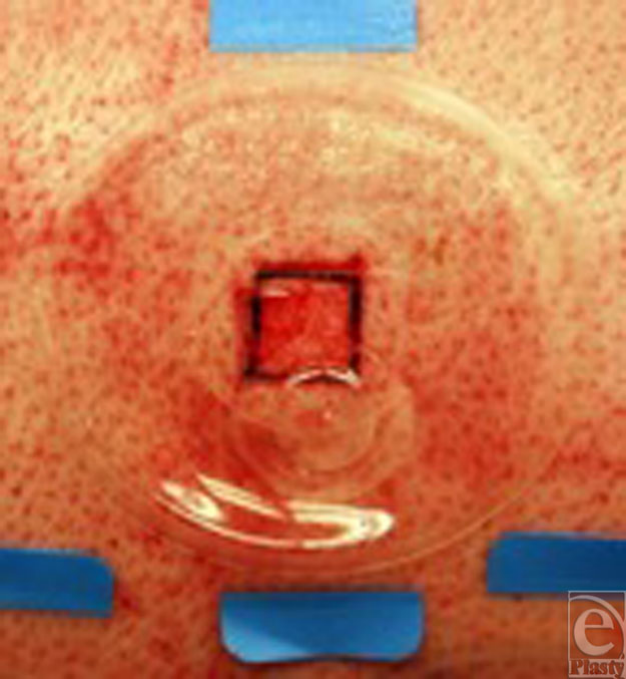
A dermatome–created partial-thickness wound, covered with a wound chamber. Close to the center of the chamber is a port, which allows multiple injections into the wound chamber.

The dorsum of the pigs was waxed (Nair, Church & Dwight, Princeton, New Jersey), shaved, and thoroughly disinfected. Twenty-four squares (15 mm × 15 mm) per pig were outlined by a tattoo machine (Special Electric Tattoo Marker; Spaulding Enterprises; Voorheesville, New York). After the skin was prepared with povidone/iodine, the skin within the tattoo borders was excised at a depth of 18/1000 of an inch using an electric dermatome (Fig 1).

For all sites, medical adhesive (Hollister, Inc, Libertyville, Illinois) was brushed onto the skin surrounding the wounds. Self-enclosed polyurethane wound chambers (Corium International, Grand Rapids, Michigan) were sealed to the surrounding skin.

Wounding of the diabetic pigs was done 14 days after induction of diabetes in the same fashion as described above.

### Maintenance of wound fluid

Wound fluid was exchanged, measured, and refilled daily, while the pigs were anesthetized with tiletamine HCl and zolazepam HCl and were suspended in a Panepinto sling. The fluid was kept on ice throughout the procedure, centrifuged at 1200 rpm, flash frozen, and stored at –70°C.

### Total protein assay

A commercial protein assay (#23227, Pierce, Rockford, Illinois) was used as described in the manufacturer's protocol. The absorbance of the resulting turbidity was read by a photospectrometer at 420 nm and compared with the protein standard similarly treated.

### Histological evaluation

Diagonal biopsies, covering the whole wound, were excised from the pigs on days 6, 8, 10, and 12. On every biopsy day, 6 wounds in each study group and 6 wounds in the control group were excised. Biopsied wounds were excluded from further study.

Specimens were paraffin-embedded, cut into 6-μm sections, and stained for hematoxylin and eosin. Moreover, the number of rete ridges per 2.5 mm of wound surface and the number of cell layers were evaluated.

The thickness of epidermis was measured in 10 random locations in the histologies from the different biopsy days.

### Statistics

Means and standard errors for healing time were calculated for the groups. Statistical significance between groups was analyzed with the nonparametric Mann-Whitney *U* test.

## RESULTS

### Healthy pigs

Using this protocol, it was possible to get a complete set of data from 70 of the 72 wounds. Because of disruption of the wound chambers by the pig, 2 wounds could not be evaluated on day 10 and consequently on day 12, one in the ACCS group every other day and one in the control group. This decreased the number of wounds in these groups on days 10 and 12 to 5 instead of 6. The pigs appeared healthy and behaved normally throughout the experiment. There was no adverse effect from the ACCS on any wound or surrounding skin at any time.

The macroscopic appearance of the wounds during the healing process was similar in all groups at each time point. There was no redness or other sign of adverse side effect in the ACCS-treated wounds. The scarring at the end point of the study was similar in all groups and all wounds.

**Figure 2:**
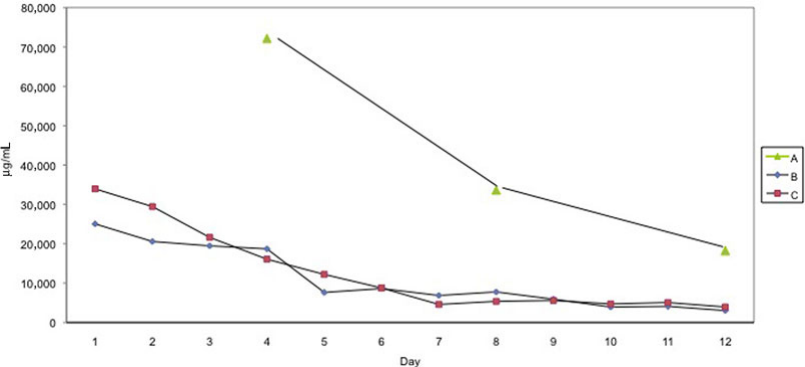
Characteristics of the protein concentration for ACCS qd and the control group based on a daily collection and for group ACCS collected every 4th day. Note that the protein in the wound fluid from the group ACCS q4d wounds has been accumulated over 4 days and has a different base line compared with group ACCS qd and the control, which accumulated over 1 day. A, ACCS q4d; B, ACCS qd; and C, control. ACCS indicates amnion-derived cellular cytokine solution.

No difference could be shown in the total protein concentration in the wound fluid between group ACCS every 4 days and the control group. All controls were used to obtain significant numbers. Group ACCS every 4 days had a much higher protein concentration due to the fact that protein was allowed to accumulate for 4 days rather than for 1 day (Fig 2).

**Figure 3:**
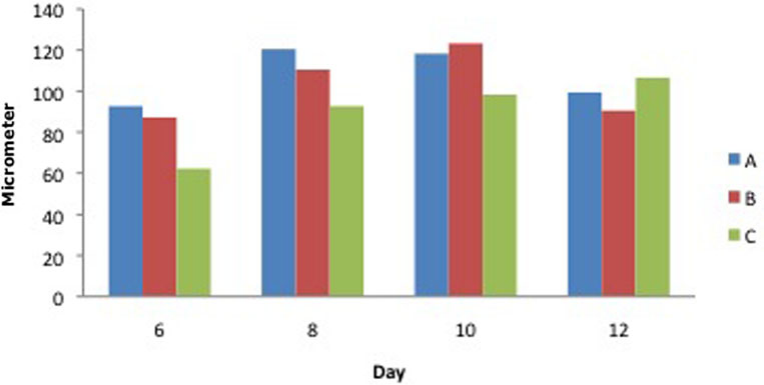
Thickness of the epidermis in histological sections on biopsy days 6, 8, 10, and 12 in healthy pigs. A, ACCS q4d; B, ACCS qod; and C, control. ACCS indicates amnion-derived cellular cytokine solution.

Measurement of thickness of the epidermis showed a statistically significant difference between the study groups (ACCS every day and ACCS every 4 days) compared with controls on day 6, 8, and 10. No difference could be observed by day 12 (Fig 3).

**Figure 4:**
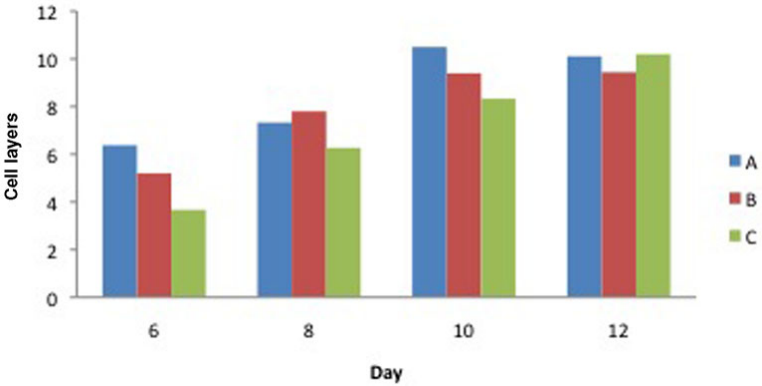
Number of cell layers on biopsy days 6, 8, 10, and 12 in healthy pigs. A, ACCS q4d; B, ACCS qd; and C, control. ACCS indicates amnion-derived cellular cytokine solution.

Figure 4 shows the number cell layers in the epidermis. This graph mirrors the results shown in Figure 3. The grid used for selecting the point of measurement was the same in both experiments but no attempt was made to measure in exactly the same place. It should also be pointed out that the cells are rounder and thicker on day 6 than on day 12, which explains why the lower numbers of cells on day 6 do not correlate with a significantly thinner epidermis.

The evaluation of rete ridges per 2.5 mm showed, except for group ACCS every 4 days on day 10, a consistently and significantly larger number of rete ridges in groups ACCS every 4 days and ACCS every day as compared with the control group (Fig 9).

### Diabetic pigs

**Figure 5:**
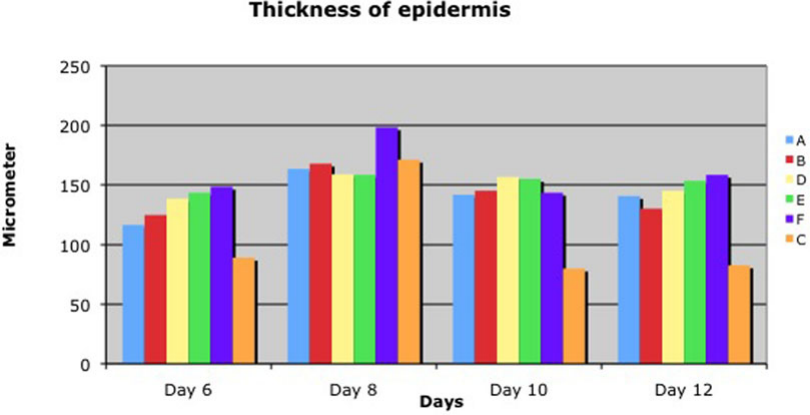
Thickness of epidermis in diabetic pigs (A: 0.455-mL ACCS/cm^2^, B: 0.255-mL ACCS/cm^2^, D: 0.111-mL ACCS/cm^2^, E: 0.045-mL ACCS/cm^2^, F: 0.022-mL ACCS/cm^2^, C: Control)

**Figure 6:**
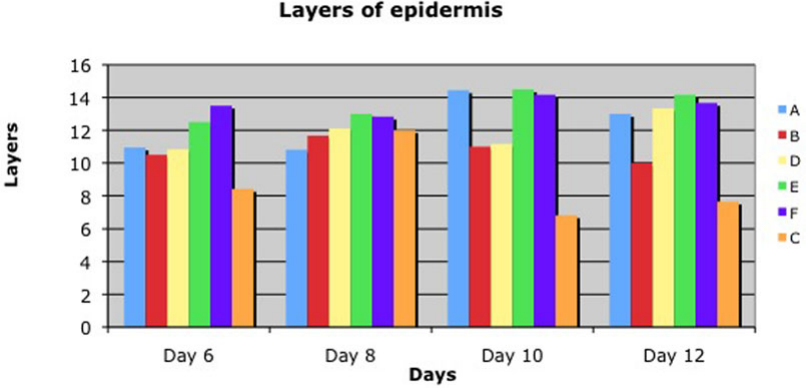
Layers of epidermis in diabetic pigs (A: 0.455-mL ACCS/cm^2^, B: 0.255-mL ACCS/cm^2^, D: 0.111-mL ACCS/cm^2^, E: 0.045-mL ACCS/cm^2^, F: 0.022-mL ACCS/cm^2^, C: control).

**Figure 7:**
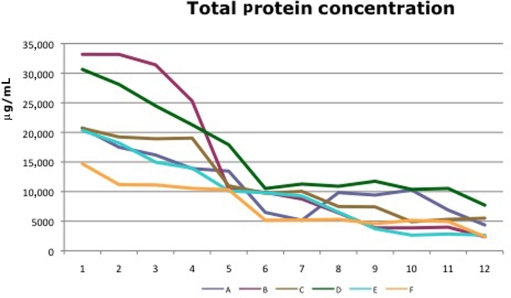
Total protein concentration in wound fluid of diabetic pigs (A: 0.455-mL ACCS/cm^2^, B: 0.255-mL ACCS/cm^2^, D: 0.111-mL ACCS/cm^2^, E: 0.045-mL ACCS/cm^2^, F: 0.022-mL ACCS/cm^2^, C: control).

**Figure 8:**
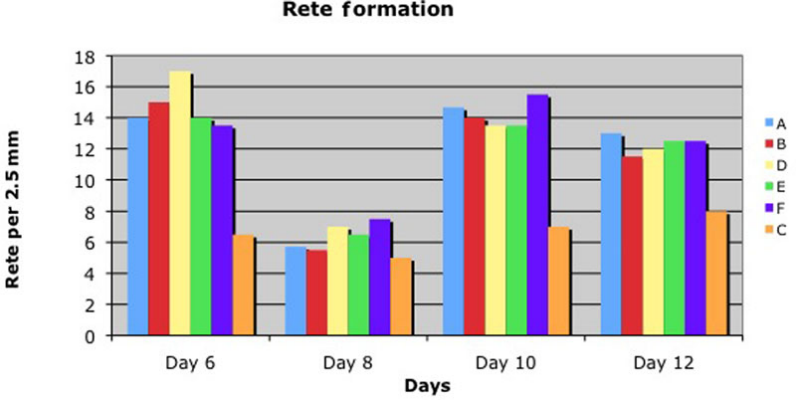
Number of rete ridges per 2.5 mm on biopsy days 6, 8, 10, and 12 in diabetic pigs (A: 0.455-mL ACCS/cm^2^, B: 0.255-mL ACCS/cm^2^, D: 0.111-mL ACCS/cm^2^, E: 0.045-mL ACCS/cm^2^, F: 0.022-mL ACCS/cm^2^, C: control).

**Figure 9:**
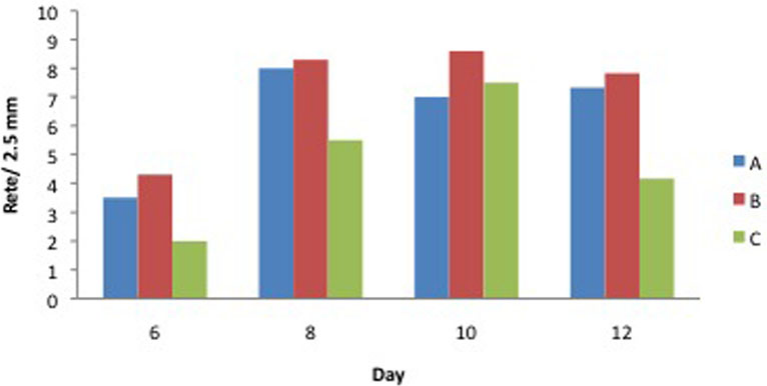
Number of rete ridges per 2.5 mm on biopsy days 6, 8, 10, and 12 in healthy pigs (A: ACCS q4d, B: ACCS qod, C: control).

Summarizing the histological data, including thickness of the epithelium, number of cell layers in the newly formed epithelium, and rete formation, we recognized numeric differences. More epidermal cell layers and thicker epidermis were found at all concentrations of ACCS compared with the saline control (Figs 5–8). Moreover, the rete formation in the ACCS groups was higher than in the control.

ELISA evaluation of the wound fluid showed an increase of the protein concentration in the first days followed by a drop between days 4 and 6. The protein concentration of the last days (7–12) remained stable (Fig 7). Similar data could be shown in the previous experiments on healthy pigs.

## DISCUSSION

**Figure 10:**
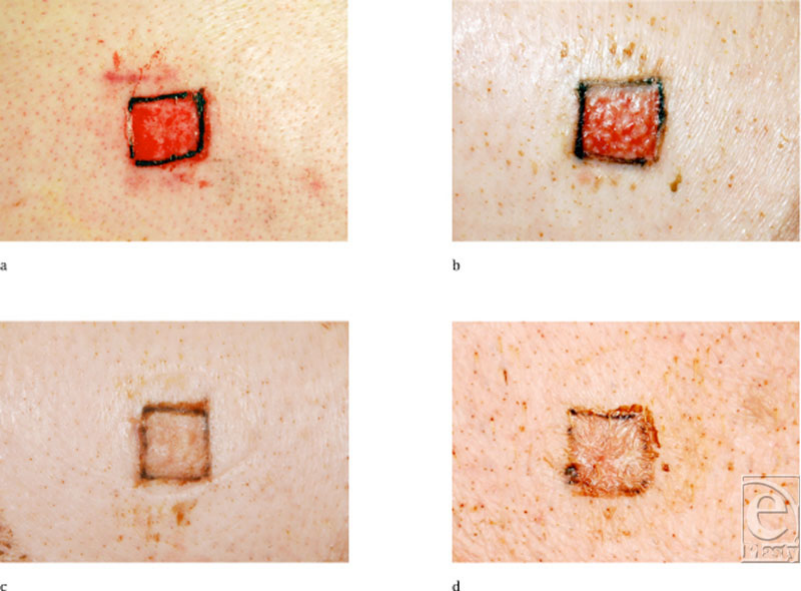
Macroscopic pictures of representative wounds on days 6 and 10 in diabetic pigs: (a) day 6, 0.455-mL ACCS/cm^2^; (b) day 6, control; (c) day 10, 0.455-mL ACCS/cm^2^; and (d) day 10, control. ACCS indicates amnion-derived cellular cytokine solution.

Cells in culture secrete factors that may provide support to or affect the growth, differentiation, and protein production of other cells.^9^ Stem cells and stem cell–like multipotent cells are known to produce cytokine growth factors that serve as mediators to the cellular processes of the wound-healing scheme.^3^ Normal wound healing is accomplished by a combination of cytokines, and they occur in a “natural” cascade.^11^ ACCS has been shown to have cytokines at physiological levels including PDGF, VEGF, angiogenin, TGF-β_2_, TIMP-1, and TIMP-2.^9^ Applying ACCS in a continuous “wet” environment accelerated and improved healing in the 2 experiments in both normal and diabetic swine. Maturation of the healed epidermis was accelerated both macroscopically (Fig 10) and microscopically.

Comparing the thickness of the epidermis, the layers of the epidermis, or the rete ridge formation in both experiments demonstrated ACCS improvement versus saline control. It did not seem to matter the frequency of application, or the dose of ACCS chosen. Even the smallest dose, 0.022 mL/cm^2^ of ACCS showed the improvement (Figs 6, 7, and 8).

There was very little difference in the healing of partial-thickness donor site wounds in the diabetic swine versus the normal swine. This is not surprising. The model presents the ideal situation for reepithelialization with a “wet” environment, chambers to prevent inflammation and/or infection, and a dermal base, which does not require keratinocyte migration. Although more than 100 physiological factors contribute to wound-healing deficiencies in individuals with diabetes, it has not been shown that reepithelialization of a partial-thickness wound in an ideal protected environment is impaired.^12^,^13^

In conclusion, ACCS has been demonstrated to accelerate and/or improve wound healing in acute and chronic wound models,^1^ mesh graft interstices,^14^ and now in partial-thickness donor site wounds. A clinical trial is now under way to evaluate its safety and efficacy in human wounds.
